# Genome-scale CRISPR screen identifies TMEM198 driving double membrane vesicle formation in swine alphacoronavirus and murine betacoronavirus infected cells

**DOI:** 10.1371/journal.ppat.1013211

**Published:** 2025-05-30

**Authors:** Lei Shi, Yu Zhang, Yueyue Duan, Maowen Sun, Cong Yuan, Liyan Cao, Xiangyu Kong, Wenlong Zhang, Haixue Zheng, Qi Wang

**Affiliations:** 1 State Key Laboratory for Animal Disease Control and Prevention, Lanzhou Veterinary Research Institute, Chinese Academy of Agricultural Sciences, Lanzhou, China; 2 College of Veterinary Medicine, Northeast Agricultural University, Harbin, Heilongjiang, China; 3 Institute of Urban Agriculture, Chinese Academy of Agricultural Sciences, Chengdu, China; 4 Chengdu National Agricultural Science and Technology Center, Chengdu, China; Loyola University Chicago Stritch School of Medicine, UNITED STATES OF AMERICA

## Abstract

COVID-19 pandemic caused by the SARS-CoV-2 which is well-publicized cross-species transmissibility. SARS-CoV-2 belongs to genus *Betacoronavirus,* several pathogenic alphacoronaviruses have shown similar patterns of emergence. Much less attention paid to host factors required for alphacoronavirus replication compared to those of betacoronaviruses. Here, we utilized a genome-wide CRISPR-Cas9-based screen to identify TMEM198 as a critical host protein for double-membrane vesicle (DMVs) formation during the replication of swine alphacoronavirus. Gene deletion of TMEM198 led to a reduction in the levels of viral infection in cells, whereas the ectopic expression of TMEM198 correspondingly resulted in an increase in infection levels. At the mechanistic level, TMEM198 directly binds to the C-terminal of nonstructural protein 3 (nsp3c) and nonstructural protein 4 (nsp4) to participate in the formation of DMVs. The first 35 amino acids at the N-terminal of TMEM198 are critical for the formation of DMVs and viral replication. Moreover, mice with a gene deletion of TMEM198 exhibit reduced susceptibility to the *Betacoronavirus* MHV. These results identify the function of TMEM198 in the formation of DMVs during the replication of swine alphacoronavirus and murine betacoronavirus.

## Author summary

The intracellular host membrane system is reorganized by coronaviruses during infection to form DMVs that facilitate viral RNA replication and transcription. Nonstructural protein 3 (nsp3) and nonstructural protein 4 (nsp4) of coronaviruses are essential for DMVs formation. However, the host factors involved in the DMVs complex remain largely unknown. In this study, we identified TMEM198 as a novel host protein that facilitates DMVs formation during coronavirus infection through an unbiased genomic CRISPR-Cas9 screening method. We found that the first 35 amino acids of the N-terminal region of TMEM198 are critical for DMVs formation and viral replication. Importantly, we demonstrated that TMEM198 is required for the replication of swine alphacoronavirus and murine betacoronavirus. Our study identifies TMEM198 as a key determinant governing the biogenesis of DMVs, thereby promoting coronavirus infection.

## 1. Introduction

The coronaviruses belong to the family *Coronaviridae*, and the name of coronaviruses is derived from the Latin word “corona” which refers to the crown-like appearance of the embedded envelope protein. Coronaviruses cause a wide range of diseases in both animals and humans. [1,2]. Its subfamily *Orthocoronavirus* is divided into four genera, *Alphacoronavirus*, *Betacoronavirus*, *Gammacoronavirus*, and *Deltacoronavirus* [3]. Coronaviruses cause respiratory, gastrointestinal, and central nervous system diseases in wildlife, livestock, and humans, thereby posing significant threats to public health and resulting in economic losses [4].

In pigs, coronaviruses are the causative agents of gastrointestinal infections, representing a critical concern from both clinical and epidemiological perspectives. Current major causative agents of lethal watery diarrhea in piglets are four conaviruses including transmissible gastroenteritis virus (TGEV), porcine epidemic diarrhea virus (PEDV), swine acute diarrhea syndrome-coronavirus (SADS) and porcine deltacoronavirus (PDCoV). Swine coronaviruses have emerged and spread throughout the global swine industry. These diseases cause substantial economic losses within the worldwide pork industry [5]. Pigs are regarded as a potential intermediate host for human coronaviruses and other mammalian coronaviruses [6]. Currently, there are no approved vaccines or antiviral drugs for swine pan-coronavirus. A more complete understanding of the cellular and molecular mechanisms that are exploited by coronaviruses could facilitate the development of antiviral strategies, thereby preparing for current and potential future outbreaks of coronaviruses.

The life cycle of coronaviruses includes entry, uncoating, translation, proteolysis, the formation of double-membrane vesicles (DMVs) for anchoring the replication-transcription complexes (RTCs), genome replication and mRNA synthesis, translation and assembly, exocytosis, and budding [7]. Among these steps, DMVs formation plays a critical role in the life cycle of coronaviruses. Coronaviruses remodel the membranes of the endoplasmic reticulum (ER) to form DMVs during viral replication [8–10]. Viral non-structural proteins are essential for the biogenesis of DMVs, which are crucial for the completion of the viral life cycle. During the viral infection, viruses commonly employ host factors to facilitate viral attachment, entry, transcription and assembly-release [11]. Currently, the cellular host factors are associated with swine coronavirus life cycle remain unclear and urgently require further in-depth research. In recent years, the application of CRISPR-Cas9 based genome-wide screening approaches has become indispensable for studying cellular factors involved in viral infection due to their specificity and reproducibility [12]. CRISPR genetic screens identify essential host factors of DENV and HCV infections [13]. Genome-wide CRISPR knockout screening identified capicua as a critical factor for cell-intrinsic immunity and the replication of influenza A viruses [14].

In this study, we generated a porcine genome-wide CRISPR knockout library in PK15 cells and subsequently subjected this PK15 knockout library to infection with the *Alphacoronavirus* TGEV. Deep sequencing analysis of the enriched single guide RNA (sgRNA) population identified numerous genes that may be involved in the replication of TGEV. Validation studies revealed that transmembrane protein 198 (TMEM198)

plays a crucial role in the formation of the DMVs. TMEM198 is a poorly understood protein that was previously found to associate with LRP6 and recruit casein kinase family proteins to phosphorylate key residues important for LRP6 activation [15]. No correlation was found between TMEM198 and viral replication in previous study, which indicates TMEM198 is a novel host proviral factor implicated in coronavirus replication in our study. Knockout of TMEM198 dramatically inhibit viral infection and delay the progression of diseases. Our research suggests that targeting TMEM198 is a very promising approach for developing broad-spectrum antiviral therapies.

## 2. Results

### 2.1. A genome-scale CRISPR screen identified host factors associated with TGEV infection

To identify the host factors involved in the replication of the *Alphacoronavirus* TGEV, we established a porcine whole-genome-scale CRISPR/Cas9 knockout (PigGeCKO) cell library based on PK15 cells and conducted a genome-wide loss-of-function screen. Prior to the screening, we induced cell death through TGEV infection at various multiplicities of infection (MOI), including 0.0001, 0.001, 0.01, and 0.1.

**Fig 1:**
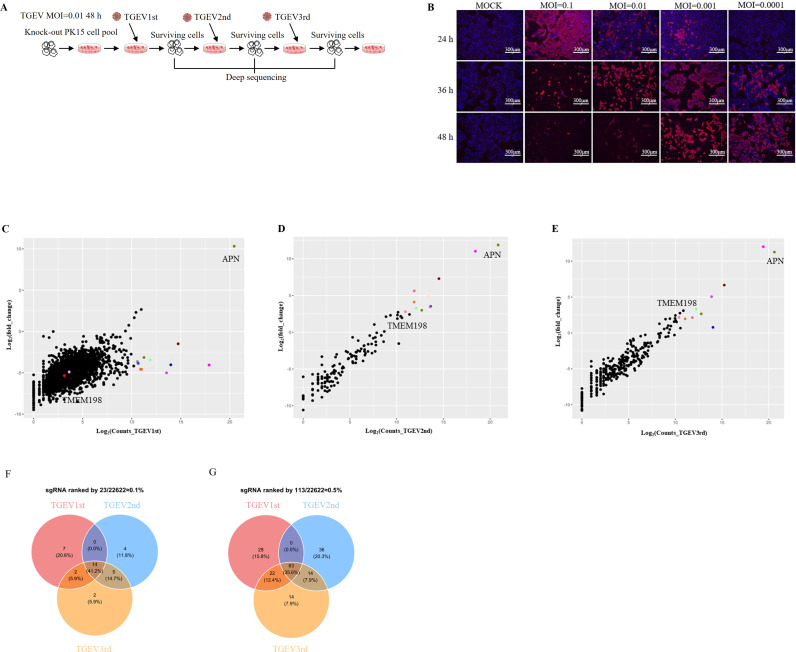
Genome-wide CRISPR screen identifies host factors associated with the *Alphacoronavirus* TGEV. **(A)** The identification of TGEV host factors utilized the porcine genome-scale CRISPR/Cas9 knockout (PigGeCKO) library. Transformed PK15-Cas9 cells were subjected to either mock treatment or TGEV challenge at a multiplicity of infection (MOI) of 0.01. Surviving cells from each round of virus challenge were isolated, followed by PCR amplification and sequencing of sgRNA. The figure was generated using resources from the OpenClipart website. **(B)** We assessed the optimal titer for TGEV-induced cell death in PK15 cells at an MOI of 0.01. TGEV-induced cell death was evaluated following infection at MOI values of 0.0001, 0.001, 0.01, and 0.1, revealing cytopathic effects for the various MOIs approximately 48h post-infection. **(C to E)** Scatter plots depict sgRNA-targeted sequence frequencies and the extent of enrichment in transformed PK15-Cas9 cells (mock-treated versus TGEV infected) across three rounds of TGEV screening. **(C)** First round. **(D)** Second round. **(E)** Third round. Counts_TGEV1st, Counts_TGEV2nd, and Counts_TGEV3rd represent the average reads from paired-end sequencing for each round. Log2 (fold change) denotes the median log2 ratio between normalized sgRNA counts of TGEV-challenged and mock-treated populations. **(F and G)** Venn diagrams illustrate the overlap in enrichment of specific sgRNA targeting sequences across the three TGEV screening rounds among the top **(F)** ~0.1% and **(G)** ~0.5% of averaged sgRNA reads. sgRNA refers to small guide RNA.

We observed cytopathic effects at different MOI approximately 48h after TGEV infection (Fig 1B). We determined that the optimal conditions for TGEV-induced cell death were an MOI of 0.01 with a 48h infection time in PK15 cells. Subsequently, we performed a screening to identify host factors that contribute to cell death under TGEV induction. The overall PigGeCKO screening strategy is illustrated in Fig 1A. We conducted three rounds of TGEV screening, using untreated stable Cas9-expressing PK15 cells (PK15-Cas9) as a negative control (Ctrl) to confirm that cell death was indeed caused by TGEV infection in each round. After 48h of TGEV infection, all PK15-Cas9 negative Ctrl cells had died, while a small number of viable cells were still observable in the PigGeCKO cell library. The surviving cells were expanded and collected for subsequent rounds of TGEV challenge. Ultimately, the integrated sgRNA cassettes from genomic DNA in the surviving mutant cells across the three rounds were amplified using polymerase chain reaction (PCR) and subjected to deep sequencing.

The screening results indicated that aminopeptidase N (APN), recognized as a functional receptor for TGEV [16,17], was significantly enriched across all three rounds of CRISPR screens (Fig 1C-1E). Among the top 0.1% of sgRNAs, several sgRNAs targeted the same gene. In the second round of challenges, two sgRNAs targeted the APN and TMEM198 genes, respectively. By comparing the enriched sgRNAs identified in the positive selection CRISPR screens, we found that 41.2% of the highly enriched sequences (≥ 0.1%) were common across the first, second, and third challenge rounds. Additionally, 35.6% of the highly enriched sequences (≥ 0.5%) were shared among all three rounds (Fig 1F and 1G). These results indicate that the candidate host factors associated with TGEV infection are reliable based on our CRISPR screening strategy.

### 2.2. TMEM198 is an essential host factor for the replication of TGEV

**Fig 2:**
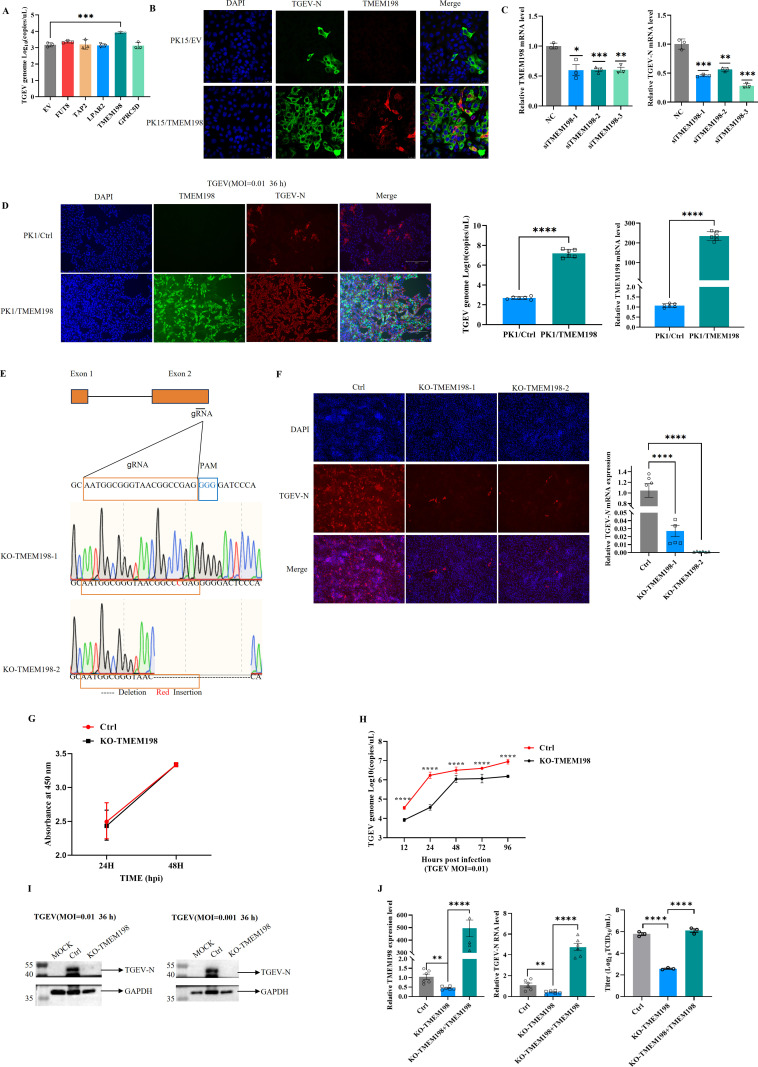
TMEM198 is a key factor required for TGEV replication. (A). The indicated host factors were overexpressed, followed by infection of the cells with TGEV at a MOI of 0.01. The absolute mRNA levels of the TGEV N gene were subsequently measured. (B) PK15 cells that overexpressed TMEM198 were infected with TGEV (MOI = 0.01), and the expression of TGEV N protein (green fluorescence) and TMEM198 (red fluorescence) was assessed via indirect immunofluorescence. (C) Following the knockdown of TMEM198 in PK15 cells, the cells were infected with TGEV (MOI = 0.01). The copy number of TGEV RNA in the infected cells was determined using RT-qPCR, normalized to actin. The Ctrl group was set to 1. (D) The detection of TGEV N protein (red fluorescence) and TMEM198 (green fluorescence) via indirect immunofluorescence in PK15 cells stably expressing TMEM198. The absolute mRNA levels of the TGEV N gene and the relative mRNA levels of the TMEM198 gene were detected by qPCR. (E) The TMEM198 knockout cell line was established, and the knockout clones were confirmed through nucleic acid sequencing. (F) Both TMEM198 knockout (KO-TMEM198) and Ctrl cells were infected with TGEV (MOI = 0.01). The expression of TGEV N protein (red fluorescence) was evaluated using indirect immunofluorescence, while the expression level of TMEM198 was measured through relative quantitative real-time PCR. (G) Cell proliferation was assessed utilizing CCK-8 assays, where Ctrl and KO-TMEM198 PK15 cells were incubated with 100 μl serum-free medium and 10 μl CCK-8 solution at 37°C for 2h, followed by measurement at a wavelength of 450 nm. (H) The KO-TMEM198 and Ctrl cells infected with TGEV at a MOI of 0.01, the viral titers were monitored at indicated time point. (I) Western blot analysis was conducted to assess the expression of the TGEV N protein in KO-TMEM198 and Ctrl cells at 36h post-infection with TGEV infection. (J) The deletion of TMEM198 was found to impair TGEV replication. The re-expression of TMEM198 in KO-TMEM198 cells restored TGEV replication. In the left panel, the relative expression level of TMEM198 under the indicated conditions was determined by RT-qPCR, normalized to actin. Ctrl group was set to 1. The middle panel, TGEV RNA copy number in the KO-TMEM198 and Ctrl cells during TGEV infection. Ctrl group was set to 1. The right panel, viral titers evaluated through virus TCID_50_ assays at 36h post viral infection. DAPI was used for nuclear staining. Error bars represent the mean ± standard deviation (SD) for triplicate experiments.

To identify key host factors that enhance TGEV replication, we overexpressed the top five enriched genes identified from the whole-genome CRISPR/Cas9 screen in PK15 cells. Following infection with TGEV (MOI = 0.01) for 36h, we quantified the absolute mRNA levels of the TGEV N gene using qPCR. Our results indicated that only TMEM198 significantly promoted TGEV replication (Fig 2A). We subsequently overexpressed TMEM198 in PK15 cells, and indirect immunofluorescence analysis demonstrated that the overexpression of TMEM198 markedly increased TGEV N protein levels (green fluorescence) (Fig 2B), thereby confirming that TMEM198 facilitates TGEV replication. In addition, knocking down TMEM198 in PK15 cells using small interfering RNA resulted in a reduction in the relative mRNA levels of the TGEV N gene during TGEV infection (Fig 2C). To rule out a nonspecific effect on cell type and confirm that the reduced viral replication levels observed upon knock-down TMEM198 is specific. Porcine IPEC-J2 intestinal epithelial cells were used to investigate TGEV replication. Knockdown of TMEM198 in IPEC-J2 cells attenuates TGEV replication (S1 Fig). We constructed a stable TMEM198-expressing cell line and subsequently infected the cells with TGEV at a MOI of 0.01 for 36h. Both indirect immunofluorescence and qPCR assays demonstrated that TMEM198 enhances TGEV replication (Fig 2D). To further investigate the role of TMEM198 in TGEV replication, we employed the CRISPR/Cas9 gene editing system to generate a TMEM198 knockout PK15 cell line. Sanger sequencing confirmed the successful establishment of the TMEM198 knockout cell line (Fig 2E). The results from immunofluorescence and qPCR analyses revealed that the levels of TGEV N protein were significantly reduced in TMEM198 knockout cells following infection with TGEV at an MOI of 0.01 (Fig 2F). To rule out a nonspecific effect on cell proliferation and confirm that the reduced viral replication levels observed upon KO-TMEM198 is specific. Cell proliferation was assessed using CCK-8 assays in TMEM198 knockout and Ctrl cells. Deletion of TMEM198 did not noticeably affect cell proliferation (Fig 2G). We observed viral titers in TMEM198 knockout cells were significantly reduced during viral infection (Fig 2H). Western blot analysis indicated that, at MOIs of 0.01 and 0.001, the TGEV-encoded N protein was either barely detectable or weakly expressed in TMEM198 knockout cells compared to Ctrl cells at 36h post-infection (Fig 2I). These findings demonstrate that the knockout of TMEM198 substantially inhibits TGEV replication. To further investigate whether TMEM198 is essential for TGEV infection, we reconstituted the TMEM198 into TMEM198 knockout cells and found that the expression of TMEM198 in TMEM198 knockout cells completely restored TGEV replication (Fig 2J). Collectively, these results suggest that TMEM198 is a critical proviral factor for TGEV infection.

### 2.3. TMEM198 enhances the transcription and replication of TGEV

**Fig 3:**
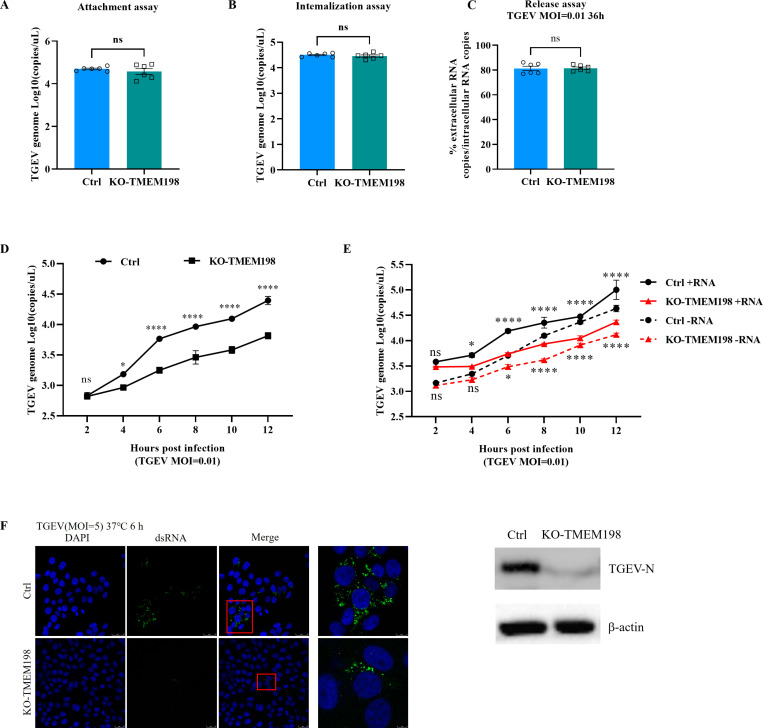
TMEM198 enhances the transcription and replication of TGEV. **(A)** KO-TMEM198 and Ctrl cells were infected with TGEV at a MOI of 5 at 4°C for 1h to assess TGEV adsorption. Viral RNA was extracted to determine the attachment of virions to the cell surface. **(B)** Following attachment of TGEV, temperature is shifted to 37 °C and internalization of viral particles is allowed to take place. The internalization of TGEV in both KO-TMEM198 and Ctrl cells was evaluated using absolute quantitative real-time PCR. **(C)** The release stage of TGEV was assessed in KO-TMEM198 and Ctrl cells infected with TGEV. The extracellular and intracellular RNA copies of TGEV were measured by RT-qPCR at 36h post-infection, and the ratio of extracellular to intracellular viral RNA copies was calculated. **(D)** KO-TMEM198 and Ctrl cells were infected with TGEV at an MOI of 0.01. The early replication of TGEV was quantified by assessing the copy number of the TGEV N gene at indicated time point using RT-qPCR. **(E)** The positive (+vRNA) or negative (−vRNA) viral RNA was quantified at the indicated time point using RT-qPCR. **(F)** Left panel, confocal microscopy to evaluate early-stage TGEV replication by detecting dsRNA in Ctrl and KO-TMEM198 cells post 6h of TGEV infection. Right panel, western blot analysis of viral protein expression at 24h post-infection. Error bars represent the mean ± standard deviation (SD) from triplicate experiments.

To explore the role of TMEM198 in the TGEV infection cycle, we examined the effects of TMEM198 on viral attachment, internalization, release and viral genome transcription. No differences were observed in viral RNA levels between Ctrl and KO-TMEM198 cells after infection with TGEV at 4°C, or following a 4°C incubation followed by 37°C incubation and proteinase K treatment. This implies that TMEM198 does not affect viral attachment and entry (Fig 3A and 3B). To further investigate the role of TMEM198 in viral assembly and release, Ctrl and KO-TMEM198 cells were infected with TGEV at a MOI of 0.01. The extracellular and intracellular RNA copies of TGEV were measured at 36h post viral infection. No statistically significant difference was detected between the ratio of extracellular RNA copies compared to intracellular RNA copies in KO-TMEM198 and Ctrl cells (Fig 3C), suggesting that TMEM198 does not modulate TGEV release.

To determine whether TMEM198 affects TGEV viral genome replication, we monitored the total viral genomic RNA (gRNA) during the early stages of infection. We observed reduced gRNA in KO-TMEM198 cells at 4h post viral infection and throughout the infection (Fig 3D). We employed strand-specific RT-qPCR to differentiate between positive-strand and negative-strand viral RNA (+vRNA and -vRNA). The production of both +vRNA and -vRNA was significantly inhibited in KO-TMEM198 cells (Fig 3E). Subsequently, we detected double-stranded RNA (dsRNA) in KO-TMEM198 and Ctrl cells. Confocal microscopy assay showed that the formation of dsRNA was dramatically suppressed in KO-TMEM198 cells at 6h post-infection. Additionally, the viral protein levels were detected in both KO-TMEM198 and Ctrl cells (Fig 3F). These results indicate that TMEM198 is a crucial proviral factor for TGEV replication.

### 2.4. TMEM198 is involved in the formation of double membrane vesicle

**Fig 4:**
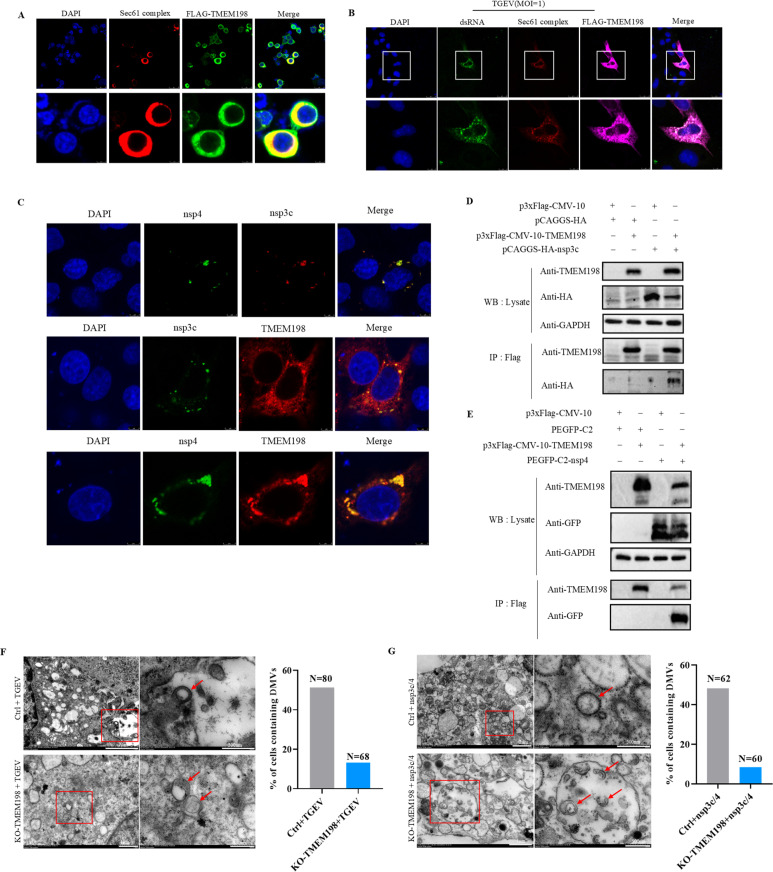
TMEM198 is involved in the formation of double membrane vesicle. **(A)** TMEM198 protein (green fluorescence) and the endoplasmic reticulum marker protein Sec61 (red fluorescence) were co-expressed in PK15 cells, and their co-localization was analyzed using confocal microscopy. **(B)** Co-localization of double-stranded RNA (dsRNA, green fluorescence), TMEM198 protein (purple fluorescence), and Sec61 was observed by confocal microscopy assay after infection with TGEV for 12h. **(C)** Co-localization analysis was performed to assess the interaction of TMEM198 with TGEV nsp3c and nsp4. **(D and E)** (Co-IP) analysis was performed to assess the interaction between TMEM198 and the viral proteins nsp3c and nsp4. **(F)** Transmission electron microscopy (TEM) analysis revealed the presence of DMVs in both Ctrl and KO-TMEM198 cells post-TGEV infection. Quantification of the percentage of cells containing DMVs relative to the total number of cells counted for each condition is provided. ‘N’ represents the number of cells counted for that condition. Scale bars are indicated as 1 μm, 2 μm, or 500 nm. **(G)** TEM analysis demonstrated the presence of DMVs in Ctrl and KO-TMEM198 cells following the overexpression of plasmids encoding nsp3c and nsp4. Quantification of the percentage of cells containing DMVs relative to the total number of cells counted for each condition is included, with ‘N’ indicating the number of cells counted for that condition. Scale bars are indicated as 1 μm, 2 μm, or 500 nm.

To further investigate how TMEM198 promotes TGEV replication in cells, we conducted a cellular localization analysis of TMEM198. The experimental results indicated that TMEM198 co-localizes with the endoplasmic reticulum marker protein Sec61 (Fig 4A). Coronaviruses transform the host endoplasmic reticulum into DMVs in infected cells [18,19]. Consequently, the confocal microscopy assay revealed that TMEM198, Sec61 and dsRNA significantly co-localize in the perinuclear region (Fig 4B). These results suggest that TMEM198 is involved in the formation of DMVs on the endoplasmic reticulum to promote TGEV replication.

Nsp3 and nsp4 of coronaviruses as the minimal components for DMVs formation which has been reported [20]. Molecular architecture of coronavirus DMVs complex showed that N-terminal domains of nsp3 are not visible in the DMVs pore complex [21]. Co-expression of C-terminal domains of nsp3 (nsp3c) and nsp4 of coronaviruses is sufficient to induce DMVs formation [22,23]. Moreover, interaction of nsp3c and nsp4 of coronaviruses was observed by immunoprecipitation analysis [22,24]. To investigate the involvement of TMEM198 in DMVs formation, we conducted a co-localization analysis of TGEV nsp3c and nsp4. Our findings indicate that these proteins co-localize in the perinuclear region of the cells (Fig 4C).

Subsequently, we conducted a co-localization analysis of TMEM198 with TGEV nsp3c and nsp4, respectively. We discovered that TMEM198 co-localizes with both nsp3c and nsp4 of TGEV in the perinuclear region of the cells (Fig 4C). To further validate the interaction between TMEM198 and nsp3c, as well as nsp4, a co-immunoprecipitation (co-IP) assay was conducted to assess the interactions of TMEM198 with both nsp3c and nsp4 of TGEV. Cells were doubly transfected with plasmids expressing Flag-TMEM198 and HA-nsp3c, and co-IP was performed using antibody to Flag tag. As shown in Fig 4D, TMEM198 interacted strongly with the nsp3c. Cells were doubly transfected with plasmids expressing Flag-TMEM198 and GFP-nsp4, and co-IP was performed using antibody to Flag tag. As shown in Fig 4E, TMEM198 demonstrated a robust interaction with nsp4. Since nsp3c and nsp4 are sufficient to rearrange the endoplasmic reticulum (ER) of host cells to form DMVs [23,25,26], we speculated that TMEM198 may play a role in DMVs formation. To test this hypothesis, we utilized transmission electron microscopy (TEM) to analyze the formation of DMVs in both KO-TMEM198 and Ctrl cells following viral infection or the overexpression of nsp3c and nsp4. We detected typical DMVs (with a diameter of approximately 200 nm) in TGEV-infected Ctrl cells. In contrast, defective DMVs were observed in KO-TMEM198 cells (Fig 4F). To further quantitation DMVs formation, we analyzed the percentage of cells containing DMVs relative to total cells by TEM during viral infection. As shown in Fig 4F, we observed significantly more DMVs in Ctrl cells infected with TGEV when compared with KO-TMEM198 cells infected with TGEV in over 50 random observation fields (Fig 4F). We also observed DMVs formation upon cells with overexpression of nsp3c and nsp4 in KO-TMEM198 cells and Ctrl cells. More DMVs were observed in Ctrl cells with overexpression of nsp3c and nsp4 when compared with KO-TMEM198 cells with overexpression of nsp3c and nsp4 (Fig 4G). The expression levels of nsp3c and nsp4 were shown in S2 Fig. All of these indicate that TMEM198 is critical for DMVs formation.

**Fig 5:**
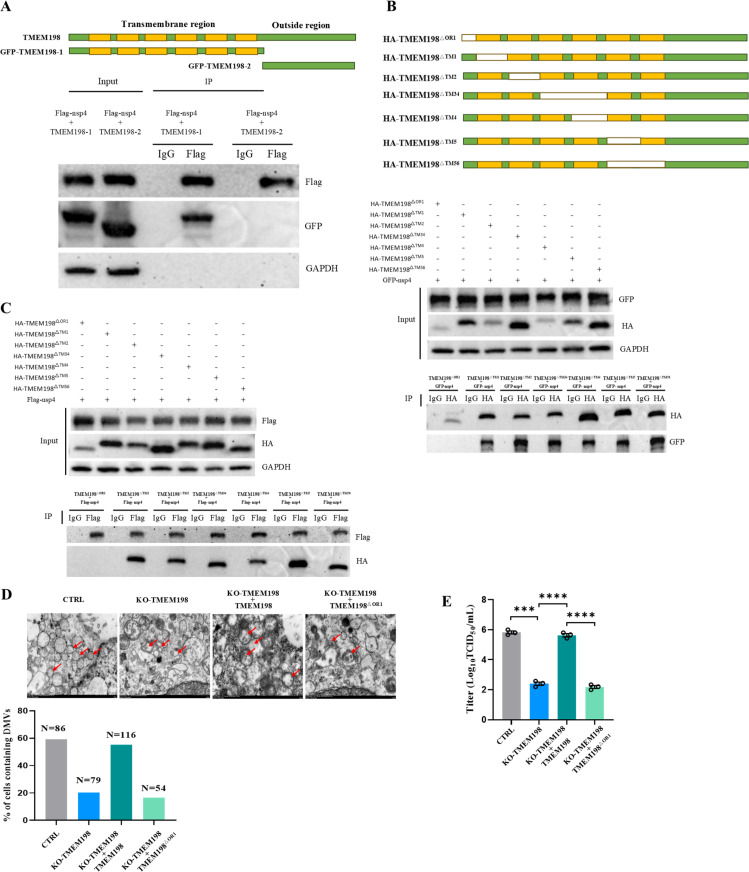
The first N-terminal 35 amino acids of TMEM198 are critical for DMVs formation and viral replication. **(A)** Schematic diagram of the TMEM198 truncations. GFP-tagged constructs were generated. Constructs were N-terminally tagged with GFP and named GFP-TMEM198-1. Constructs were C-terminally tagged with GFP and named GFP-TMEM198-2. The orange segments represent the transmembrane regions of TMEM198. HEK-293T cells were transfected with plasmids encoding indicated truncations and Flag-nsp4. Cell extracts were prepared 24h after transfection, followed by immunoblotting and IP-western analysis with indicated antibodies. **(B)** Schematic diagram of the TMEM198-1 truncation library. HA-tagged constructs were generated. Construct with deletion of the first N-terminal 35 amino acids is named HA-TMEM198^^△^OR1^. Construct with deletion of 36-61 amino acids residues is named HA-TMEM198^^△^TM1^. Construct with deletion of 62 to 89 amino acids residues is named HA-TMEM198^^△^TM2^. Construct with deletion of 90 to 142 amino acids residues is named HA-TMEM198^^△^TM34^. Construct with deletion of 116 to 142 amino acids residues is named HA-TMEM198^^△^TM4^. Construct with deletion of 143 to 174 amino acids residues is named HA-TMEM198^^△^TM5^. Construct with deletion of 143 to 197 amino acids residues is named HA-TMEM198^^△^TM56^. HEK-293T cells were transfected with plasmids encoding indicated truncations and GFP-nsp4. Cell extracts were prepared 24h after transfection, followed by immunoblotting and IP-western analysis with indicated antibodies. **(C)** HEK-293T cells were transfected with plasmids encoding indicated truncations and Flag-nsp4. Cell extracts were prepared 24h after transfection, followed by immunoblotting and IP-western analysis with indicated antibodies. **(D)** TEM analysis shows that DMVs are observed in Ctrl and KO-TMEM198 cells 12h after TGEV infection. Re-expression of TMEM198 in KO-TMEM198 cells restored DMVs formation. Re-expression of HA-TMEM198^^△^OR1^ in KO-TMEM198 cells abrogates DMVs formation. Quantification indicating the percentage of cells containing DMVs relative to total cells counted for each condition. ‘N’ indicates the number of cells counted for that condition. **(E)** Assessment of TGEV replication in Ctrl and KO-TMEM198 cells. Viral titers were evaluated by virus TCID50 assays at 36h after TGEV infection. Re-expression of TMEM198 in KO-TMEM198 cells restored TGEV replication. Re-expression of HA-TMEM198^^△^OR1^ in KO-TMEM198 cells abrogates TGEV replication.

To further explore the functional domain of TMEM198 for contributing to DMVs formation and viral replication. GFP-tagged TMEM198 truncations were generated. HEK-293T cells were transfected with different plasmid encoding truncation and Flag-nsp4. We found N-terminally tagged with GFP and named GFP-TMEM198-1 interacts with nsp4 (Fig 5A). To explore more specific function domain. HA-tagged TMEM198-1 truncation library were generated. HEK-293T cells were transfected with different plasmid encoding truncation and plasmid expressing nsp4, TMEM198 with deletion of the first N-terminal 35 amino acids (HA-TMEM198^^△^OR1^) abolished the ability of TMEM198 to interact with nsp4 (Fig 5B and 5C). Moreover, re-expression of TMEM198 in KO-TMEM198 cells restored DMVs formation and viral replication. Re-expression of HA-TMEM198^^△^OR1^ in KO-TMEM198 cells abolished the ability of TMEM198 to restore DMVs formation and viral replication (Fig 5D and 5E). All of these indicate the first N-terminal 35 amino acids of TMEM198 are critical for DMVs formation and viral replication.

### 2.5. TMEM198 is a proviral factor for swine alphacoronavirus and murine betacoronavirus

**Fig 6:**
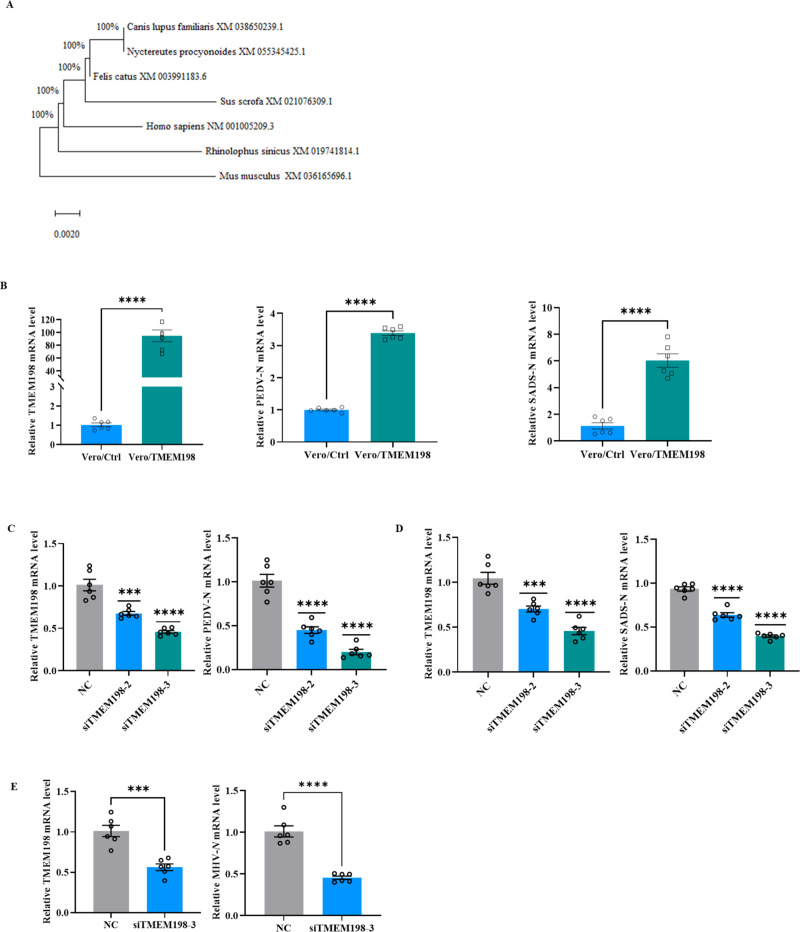
TMEM198 serves as a critical host factor essential for the replication of swine alphacoronavirus and murine betacoronavirus. (A) The alignment of the TMEM198 amino acid sequences from various species, including Sus scrofa, Homo sapiens, Mus musculus, Canis lupus familiaris, Felis catus, Nyctereutes procyonoides, and Rhinolophus sinicus, was conducted using multiple sequence alignment software (MEGA). The NCBI protein database accession numbers for TMEM198 are as follows: XM 021076309.1 for Sus scrofa, XP 001005209.3 for Homo sapiens, XP 036165696.1 for Mus musculus, XM 038650239.1 for Canis lupus familiaris, XM 003991183.6 for Felis catus, XM 055345425.1 for Nyctereutes procyonoides, and XM 019741814.1 for Rhinolophus sinicus. (B) TMEM198-expressing Vero cells were infected with PEDV and SADS at a MOI of 0.1 for 36h. The relative mRNA levels of the N gene for both PEDV and SADS were quantified using qPCR. (C) Knockdown of TMEM198 in Vero cells attenuates PEDV replication. Left panel, siRNA-mediated knockdown of TMEM198 in Vero cells and confirmed by RT-qPCR. Right panel, Vero cells were then infected with PEDV and viral replication was analyzed by viral mRNA levels determined by RT-qPCR. (D) Knockdown of TMEM198 in Vero cells attenuates SADS replication. Left panel, siRNA-mediated knockdown of TMEM198 in Vero cells and confirmed by RT-qPCR. Right panel, Vero cells were then infected with SADS and viral replication was analyzed by viral mRNA levels determined by RT-qPCR. (E) Knockdown of TMEM198 in murine L929 cells cells attenuate MHV replication. Left panel, siRNA-mediated knockdown of TMEM198 in murine L929 cells and confirmed by RT-qPCR. Right panel, murine L929 cells were then infected with MHV and viral replication was analyzed by viral mRNA levels determined by RT-qPCR.

We analyzed the amino acid sequences of TMEM198 from various species, including Sus scrofa, Homo sapiens, Mus musculus, Canis lupus familiaris, Felis catus, Nyctereutes procyonoides, and Rhinolophus sinicus. Notably, the protein sequences of TMEM198 exhibit high conservation (Fig 6A). Furthermore, we examined the amino acid sequences of nsp3 and nsp4 from porcine coronaviruses (TGEV, PEDV, and SADS) as well as MHV. The similarity of nsp3 exceeds 70%, while the similarity of nsp4 surpasses 90% (S3 Fig). Based on high similarity of amino acid of TMEM198 across different species and nsp3/nsp4 from various coronaviruses, we hypothesize that TMEM198 may serve as a broad-spectrum host factor involved in the infection processes of multiple coronaviruses. Subsequently, we infected Vero cells stably expressing TMEM198 with PEDV and SADS, respectively. The qPCR results indicated that TMEM198 can enhance the replication of both PEDV and SADS (Fig 6B). Additionally, we evaluated PEDV and SADS infection in TMEM198 knockdown Vero cells. As shown in Fig 6C, the knockdown of TMEM198 in Vero cells significantly reduces the replication of PEDV. Similarly, Fig 6D demonstrates that the knockdown of TMEM198 in Vero cells also diminishes the replication of SADS. We subsequently examined MHV infection in TMEM198 knockdown murine L929 cells. As presented in Fig 6E, the knockdown of TMEM198 in murine L929 cells leads to a notable reduction in MHV replication. These findings indicate that TMEM198 serves as a critical host factor essential for the replication of swine alphacoronavirus and murine betacoronavirus.

### 2.6. TMEM198 is required for murine betacoronavirus infection in vivo

**Fig 7:**
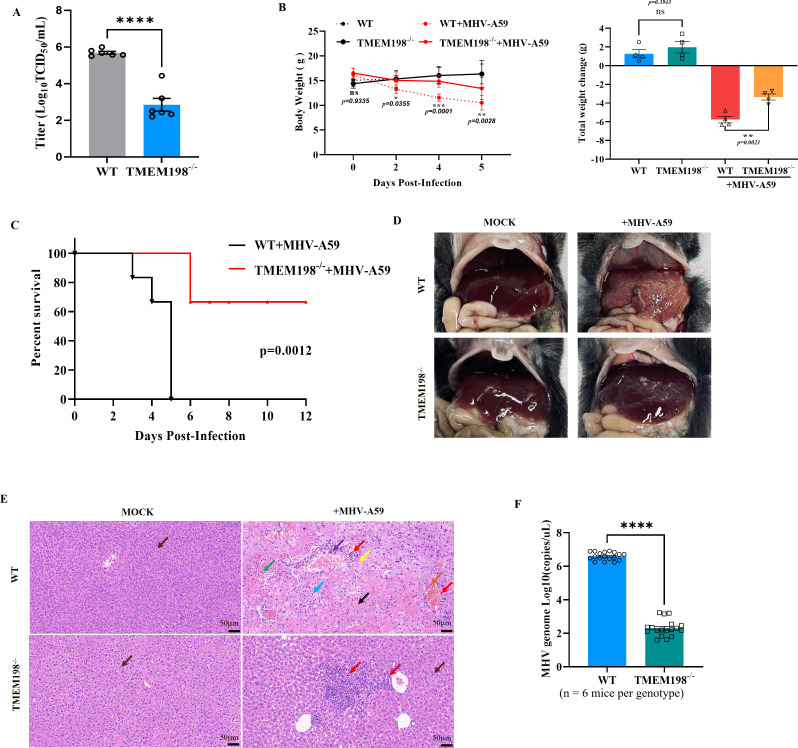
TMEM198 is required for murine betacoronavirus infection in vivo. **(A)** At 5 days post-infection, virus titers in liver tissue were evaluated using TCID_50_ assays (n = 6). **(B)** Left panel, line graphs showing the body weight of TMEM198^−/−^ and WT mice during the 5 days post-inoculation with MHV (n = 8 mice per genotype). Right panel, the weight loss of wild type and KO-TMEM198 mice at day 5 post-inoculation of MHV was analyzed. **(C)** Survival curves for TMEM198^−/−^ and WT mice infected with MHV (n = 8 mice per genotype). **(D)** Gross postmortem examination of liver tissue in TMEM198^−/−^ and WT mice infection with MHV at day 5 post-inoculation of MHV. **(E)** Histopathological analysis of the degree of liver damage in TMEM198^−/−^ and WT mice infection with MHV at day 5 post-inoculation of MHV. The histological examination was performed on liver tissue samples stained with hematoxylin and eosin **(H&E)**. **(F)** Viral copies in liver tissue were evaluated using qPCR to detect the MHV N gene in the TMEM198^−/−^ and WT mice at day 5 post-inoculation of MHV.

To determine whether the knockout of TMEM198 affects the replication capacity of coronavirus in vivo. TMEM198 homozygous knockout (TMEM198^−/−^) mice alongside wild-type (WT) mice to conduct a challenge experiment with murine hepatitis virus (MHV). Approximately 4-week-old TMEM198^−/−^ and WT mice were intraperitoneally injected with MHV at a concentration of 1.5 × 10^5^ plaque-forming units (p.f.u.). Ctrl groups received an equal volume of Dulbecco’s Modified Eagle Medium (DMEM) via the same route. We assessed the viral titer in the liver tissue of both WT and TMEM198^−/−^ mice at 5 days post-infection. As shown in Fig 7A, TMEM198^−/−^ mice exhibited significantly reduced viral titers compared to WT mice. Daily monitoring of body weight was performed, and lethality among the mice was recorded. Notably, TMEM198^−/−^ mice experienced less weight loss compared to their WT counterparts (Fig 7B). The weight loss of WT and TMEM198^−/−^ mice on day 5 post-inoculation with MHV is analyzed in the right panel of Fig 7B.

We found that TMEM198^−/−^ mice exhibited a significantly delayed progression of disease following MHV infection compared to WT mice (Fig 7C). Moreover, TMEM198^−/−^ mice showed a marked reduction in liver tissue damage when compared to the livers of WT mice infected with MHV (Fig 7D). Histopathological analysis revealed extensive areas of necrosis in the livers of WT mice, characterized by severe lesions that occupied the entire organ (Fig 7E). The liver cord structure was disordered, displaying widespread hepatocyte necrosis (indicated by black arrows), homogeneous red staining in the cytoplasm, extensive hemorrhage (green arrows), and a small amount of scattered lymphocyte infiltration (red arrows). A few hepatocytes exhibited ballooning degeneration (blue arrows), characterized by swollen cells resembling balloons, with centralized nuclei and sparse reticular structures in the cytoplasm. Additionally, a small number of hepatocytes displayed fatty degeneration (yellow arrows), evidenced by the presence of round cytoplasmic vacuoles. Notably, there was significant vascular congestion and dilation (orange arrows), with white blood cells present within the vessels, and occasional endothelial cell necrosis and shedding (purple arrows), accompanied by nuclear fragmentation. In contrast, the livers of TMEM198^−/−^ mice exhibited slight necrotic lesions (Fig 7E), with milder granular degeneration of hepatocytes in the observed field (brown arrows) and focal infiltration of lymphocytes and granulocytes (red arrows). The assessment of viral load in the livers of TMEM198^−/−^ and WT mice revealed that the viral titers in the livers of TMEM198^−/−^ mice were significantly lower than those in WT mice (Fig 7F). These results suggest that mice with a gene deletion of TMEM198 exhibit reduced susceptibility to the *Betacoronavirus* MHV.

## 3. Discussion

Currently, swine coronaviruses including PEDV, TGEV, SADS, and PDCoV pose significant threats to the pig industry. Some of these viruses have the potential to infect humans, raising public health concerns. Previous studies have shown that PDCoV can cross the species barrier from pigs to humans, which poses a risk particularly to young children [27]. Although SADS-CoV has not yet been documented to infect humans, it exhibits broad cellular tropism and potential for cross-species transmission. Reports indicate that SADS can infect various primary human respiratory and intestinal cells [28], highlighting possible public health implications.

Pigs serve as intermediate hosts in wildlife-to-human spillover events, positioning gene-edited antiviral pigs as promising candidates for novel strategies aimed at combating viral transmission [29]. The CRISPR/Cas9 technology has emerged as the preferred method due to its low technological barrier and high efficiency [30]. Pigs with CD163 depletion via the CRISPR/Cas9 system exhibit resistance to multiple PRRSV isolates [31]. Additionally, pigs expressing antiviral small hairpin RNAs (shRNAs) integrated at the porcine Rosa26 locus demonstrate resistance to CSFV infection [32]. APN-KO pigs generated through CRISPR/Cas9 technology have been reported to resist TGEV infection [33]. Despite extensive research on antiviral pigs, the long-term effects of commercially approved antiviral pigs remain uncertain. Notably, the FDA’s approval of GGTA1 gene-KO pigs offers significant promise for the commercialization of antiviral pigs.

CRISPR/Cas9 system is currently the most widely utilized gene editing system. The sgRNA lentiviral library, developed within the framework of CRISPR/Cas9, functions as an effective screening tool and has been documented for its utility in identifying host factors that are critical for various viral infections. Notable examples include SARS-CoV-2 [34–38], JEV [39], MERS-CoV [37], SARS-CoV [40], IAV [14,41] and HIV [42]. These studies demonstrate the reliability and superiority of this system. In this study, we aimed to identify the critical host factors associated with swine coronavirus infection by designing 95,710 specific sgRNAs targeting 32,614 genes within the porcine genome. We identified TMEM198 as a proviral host factor involved in the the replication of swine alphacoronavirus and the *Betacoronavirus* MHV *in vitro*. We also observed that infection with MHV in TMEM198^−/−^ mice significantly delayed disease progression and markedly reduced viral titers (Fig 7). TMEM198 is a member of the transmembrane (TMEM) protein family, officially designated as Transmembrane Protein 198. Our study is the first report to demonstrate the functional correlation between TMEM198 and viral infection.

To further elucidate the mechanism by which TMEM198 promotes TGEV replication, we conducted a subcellular localization analysis of TMEM198. The results suggest that TMEM198 is localized within the endoplasmic reticulum of the cells. In the absence of TMEM198, TGEV or nsp3c/4 induces defective DMVs. The reconstitution of TMEM198 in KO-TMEM198 cells recovers DMVs formation and viral replication. The reconstitution of TMEM198 with deletion of the first N-terminal 35 amino acids named TMEM198^^△^OR1^ abrogates DMVs formation and viral replication in KO-TMEM198 cells (Fig 5). All of these indicate that TMEM198 is essential for the formation of DMVs and the replication of coronaviruses.

Viruses exploit cellular resources to facilitate their replication. Positive-sense single-stranded RNA (+ssRNA) viruses remodel intracellular membranes into specialized structures that support viral replication. Various viruses, including coronaviruses, enteroviruses, noroviruses, and hepatitis C virus, have been found to induce DMVs. Hepatitis C virus (HCV) is reported to induce significant remodeling of membranes primarily derived from the endoplasmic reticulum [30]. Human noroviruses induce the formation of replication complexes, with the nsp4 of human noroviruses being the most critical single determinant [43]. Coronaviruses induce the formation of DMVs [44–47], which create a large network associated with viral replication and transcription [18,48,49]. This network isolates the coronavirus RNA from the rest of the cellular environment, facilitating the replication of the coronavirus genome.

We demonstrated that TMEM198 significantly promotes the replication of swine alphacoronavirus and murine betacoronavirus *in vitro*. In addition, TMEM198 also enhances the replication of the *Betacoronavirus* MHV in mice. Here, TMEM198^−/−^ and wild-type mice (approximately 4 weeks old) were intraperitoneally injected with MHV. The outcome of MHV infection of 4- to 6-week-old C57BL/6 mice depends on the dose and the route of inoculation [50]. After MHV infection, TMEM198^−/−^ mice demonstrated a significant delay in the onset of disease. In comparison to the livers of MHV-infected wild-type mice, those of TMEM198^−/−^ mice exhibited a marked reduction in liver tissue necrosis and damage induced by MHV infection. Furthermore, the viral load in the livers of TMEM198^−/−^ mice was significantly lower than that observed in WT mice. These findings suggest that TMEM198 plays a critical role in the infection process of the coronavirus MHV *in vivo*.

In summary, we have identified and validated TMEM198 as a proviral host factor for swine alphacoronavirus and murine betacoronavirus infections via genome-scale CRISPR screen. Our results emphasize the potential for developing broad-spectrum antiviral drugs that target TMEM198 to inhibit both current and emerging coronaviruses, demonstrating the significant potential of TMEM198 as a therapeutic target for coronavirus treatment.

## 4. Materials and methods

### Ethics statement

All experiments involving mice were conducted in accordance with the recommendations outlined in the ‘Guidelines for the Care and Use of Laboratory Animals’ issued by the Ministry of Science and Technology of China. These experiments received approval from the Animal Ethics Committee of the Lanzhou Veterinary Research Institute, Chinese Academy of Agricultural Sciences (License no. LVRIAEC-2024–070). Furthermore, the protocols for animal care and maintenance adhered to the guidelines established in the ‘Regulations on the Management of Laboratory Animals’ by the Ministry of Science and Technology of China.

### Plasmid construction

To construct the lentiviral sgRNA expression vector, the lentiCRISPR V2 vector was digested using the BsmBI restriction enzyme. Paired sgRNA oligonucleotides were annealed and subsequently cloned into the linearized vector. For the rescue experiment, the overexpression vector was constructed by cloning the coding sequence of TMEM198 into the p3xFLAG-CMV-10 vector (Invitrogen), which had been linearized with EcoRI and KpnI restriction enzyme. Additionally, to generate TMEM198 stable cell lines, the coding sequence of TMEM198 was cloned into the pLVX-IRSE-Puro vector, which was linearized using EcoRI and XhoI restriction enzyme.

### Cells, viruses and reagents

PK15, PK1, L929, and 293T cells are preserved in our laboratory. PK1 cells were cultured in M199 medium supplemented with 10% fetal bovine serum (Gibco, USA), penicillin (100 U/mL), and streptomycin (100 μg/mL). The other cell lines were cultured in Duchenne-modified Eagle Medium (DMEM) (Gibco, USA), also containing 10% fetal bovine serum (Gibco, USA), penicillin (100 U/mL), and streptomycin (100 μg/mL). All of these cells were incubated at 37 °C with 5% CO_2_. All cells were transfected using JetPRIME (PolyPlus) reagent according to the manufacturer’s instructions. Cells were transfected with plasmid DNA once cells reached 80% confluence. 24h post-transfection, the cells were incubated with TGEV. The viruses utilized in this study include TGEV HLJ-17 (GenBank accession no. MT522161.1), MHV (GenBank accession no. MF618253.1), PEDV CV777 (GenBank accession no. AF353511.1), and SADS-CoV strain GDS04 (GenBank accession no. MF167434). MHV was generously provided by Prof. Guiqing Peng from Huazhong Agricultural University. TGEV is known to replicate in PK1 cells, whereas PEDV and SADS-CoV replicate in Vero cells, and MHV replicates in L929 cells.

### Construction of the porcine whole-genome-scale CRISPR/Cas9 knockout (PigGeCKO) cell library

The construction of the porcine genome-scale CRISPR/Cas system was based on a previously described method [39]. In brief, to establish porcine genome-wide CRISPR Knockout (GeCKO) screening sgRNA libraries, 3–5 sgRNAs were designed for each coding gene, lncRNA, or miRNA of the porcine genome. All 95,710 specific sgRNAs (targeting 32,614 porcine genes) and 1,000 non-targeted Ctrl sgRNAs were synthesized by Genewiz Corporation (Suzhou, China). We then electrically transformed the plasmid library into Endura electrocompetent cells (Biosearch Technologies). To achieve sufficient coverage, parallel transformations were performed, counting the number of colonies to reach 500-times total sgRNAs in the library. Subsequently, sgRNA library plasmids were extracted using Plasmid Plus Maxi Kit (QIAGEN, Germany) and sequenced using Illumina HiSeq2000 platform (GENEWIZ, China).

### RT-qPCR

Total cellular RNA was extracted from the cell suspension using Trizol (RNAiso Plus, Takara). Reverse transcription was performed using PrimeScript RT Master Mix (Takara) in a total volume of 10 μl. Each RT-qPCR reaction consisted of 10 μl of 2X Power SYBR Green PCR Master Mix (Vazyme), 2 μl of cDNA (100 ng), and primers at a final concentration of 400 nmol/L. The PCR protocol commenced with an initial denaturation step at 95°C for 2 minutes, followed by 40 cycles consisting of denaturation at 95°C for 15 seconds and annealing/extension at 60°C for 30 seconds. Relative expression was quantified using the 2^−^△△^Ct^ method, with the porcine β-actin gene serving as a normalization control. For absolute RT-qPCR, approximately 500 ng of viral RNA was used as a template for cDNA synthesis. Absolute RT-qPCR was conducted using SYBR Green Mix and TGEV N gene-specific primers, with a final reaction volume of 10 μl. The cDNA sequence encoding the TGEV N protein, obtained from GenBank, was cloned into the pEGFP-C2 vector to serve as an internal reference for quantifying TGEV copy number.

### Construction of a TMEM198 knockout cell line and a stable expression cell line

The TMEM198-targeting sgRNA sequence was cloned into the lentiCRISPR V2 plasmid and subsequently transfected into 293T cells alongside helper plasmids (psPAX2 and pMD2.G) to generate lentivirus. The CRISPR-Cas9 lentiviral vector, lentiCRISPR V2, was procured from Addgene. The sgRNA sequence utilized for targeting TMEM198 is AATGGCGGGTAACGGCCGAG. Lentiviruses for CRISPR were produced through CaCl_2_-BES transfection of 293T cells. One million PK15 cells were transduced with the lentiviruses and, after three days, selected with 1 µg/mL puromycin for seven days. The knockout (KO) clones were validated via DNA sequencing. Following puromycin selection, a KO-TMEM198 monoclonal cell line was established through limited dilution and confirmed via Sanger sequencing. For the establishment of TMEM198 stable cell lines, the constructed pLVX-IRSE-Puro-TMEM198 plasmids were transfected into HEK293T cells in conjunction with helper plasmids (psPAX2 and pMD2.G) to generate lentivirus. The cells were screened with puromycin and confirmed through indirect immunofluorescence.

### siRNA knockdown

The siRNA targeting TMEM198 and the negative Ctrl siRNA (NC-siRNA) were transfected separately into PK15 cells at a concentration of 50 nM using the JetPRIME (PolyPlus) transfection reagent. All siRNAs were designed and synthesized by GenePharma (Shanghai, China). At 36h post-transfection, the mRNA levels of TMEM198 were measured using RT-qPCR to evaluate knockdown efficiency.

### Virus adsorption and internalization assays

Ctrl and TMEM198 cells were infected with TGEV at a MOI of 5, followed by incubation at 4°C for 1h. Subsequently, the cells were thoroughly washed with cold phosphate-buffered saline (PBS) to eliminate unabsorbed virions, and then harvested for total RNA extraction to quantify the adsorbed TGEV virions. To assess the effect of TMEM198 on the internalization of TGEV, both Ctrl and TMEM198 cells were infected with TGEV (MOI = 5) and incubated at 4°C for 1h. The cells were washed with cold PBS to remove unattached virus particles, subsequently incubated at 37°C for 1h to facilitate the internalization of virus particles, and then treated with protease K to eliminate any remaining virus particles on the surface. Cells were harvested for RNA extraction to quantify the internalized TGEV virions.

### Confocal microscopy

Following the growth of cells on a 12-well cell culture plate, the cells were infected with TGEV at a MOI of 0.01 for 36h. Subsequently, the cells were fixed with 4% formaldehyde at room temperature for 15 minutes and permeabilized with cold 0.5% Triton X-100 in phosphate-buffered saline (PBS) for an additional 15 minutes. The cells were then incubated overnight at 4°C with either TGEV N antibodies or anti-double-stranded RNA antibodies (SCICONS, 10010200, diluted 1:1,000). Alexa Fluor 594 Goat Anti-Mouse IgG (H + L) (Invitrogen, A-11005, diluted 1:1,000), Alexa Fluor 488 Goat Anti-Mouse IgG (H + L) (Invitrogen, A-11001, diluted 1:1,000), Alexa Fluor 594 Anti-Rabbit IgG (H + L) (Invitrogen, A-11012, diluted 1:1,000) and Alexa Fluor 488 Goat Anti-Rabbit IgG (H + L) (Invitrogen, A-11008, diluted 1:1,000) were used. Nuclei were stained with 4’,6-diamidino-2-phenylindole (DAPI) (Sigma, D9542) at room temperature in the dark for 10 minutes. Observations were conducted using fluorescence microscopy (Thermo Fisher Scientific EVOS FL Auto) or Leica laser confocal microscopy (TCS SP8).

### Analysis of immunoprecipitation and immunoblotting

Cells were lysed using NP-40 lysis buffer (P0013F, Beyotime). Protein samples were prepared by adding SDS-PAGE loading buffer (5X) (P0015, Beyotime). The proteins were then separated by SDS-PAGE and transferred to a nitrocellulose membrane. The membrane was blocked with 5% defatted milk powder (prepared with 0.1% Tween-20 (P9416, Sigma) in PBS) for 1h and incubated overnight at 4°C with primary antibodies. The GAPDH antibody (Proteintech, 1E6D9, diluted 1:5,000) was used as an internal loading control. Immunoblotting signals were captured and visualized using the WesternBright ECL kit (K-12045-D50, Advansta).

293T cells were seeded in 6 cm dishes and transfected with FLAG-tagged TMEM198 along with other labeled proteins. After 36h, the cells were washed with cold PBS and lysed using NP-40 lysis buffer. The cell lysates were subjected to ultrasonic centrifugation at 4°C (12,000 g for 10 minutes) and then incubated with anti-tag magnetic beads on a roller overnight at 4°C. The magnetic beads were subsequently washed five times with lysis buffer, and the bound proteins were analyzed by Western blotting.

### Quantification and statistical analysis

All statistical analysis was performed with unpaired Student’s t-test, and are considered significant when the p-value is less than 0.05. *, **, *** and **** indicate p-values of less than 0.05, 0.01, 0.001 and 0.0001 respectively. n.s = not significant. The number of times for DMVs observation experiment is repeated for three times.

## Supporting information

S1 Fig Knockdown of TMEM198 in IPEC-J2 cells attenuates TGEV replication. Left panel, siRNA-mediated knockdown of TMEM198 in IPEC-J2 cells and confirmed by RT-qPCR. Right panel, IPEC-J2 cells were then infected with TGEV and viral replication was analyzed by viral mRNA levels determined by qRT-PCR.
(PPTX)

S2 Fig Nsp3c and nsp4 expression verified by Western blot at 24h after transfection. (PPTX)

S3 Fig Phylogenetic relationships between nsp3 and nsp4 of different coronavirus. (A) Phylogenetic relationships between nsp3 of different coronavirus. (B) Phylogenetic relationships between nsp4 of different coronavirus. These phylogenetic trees were constructed using the maximum likelihood method in Mega11 software.
(PPTX)

S1 Data Data that underlies this paper. Excel spreadsheet containing, in separate sheets, the underlying numerical data for Figs 2H, 2J, 3A, 3B, 3C, 3D, 3E, 5E, 6B, 6C, 6D, 6E, 7A, 7B, 7F and S3.
(XLSX)
