# Supplementary figures and images for: Genome-scale CRISPR screen identifies TMEM198 driving double membrane vesicle formation in swine alphacoronavirus and murine betacoronavirus infected cells
Source: PLoS Pathog. 2025 May 30;21(5):e1013211. doi: 10.1371/journal.ppat.1013211 (PMC12157921; doi:10.1371/journal.ppat.1013211)

## Slide 1
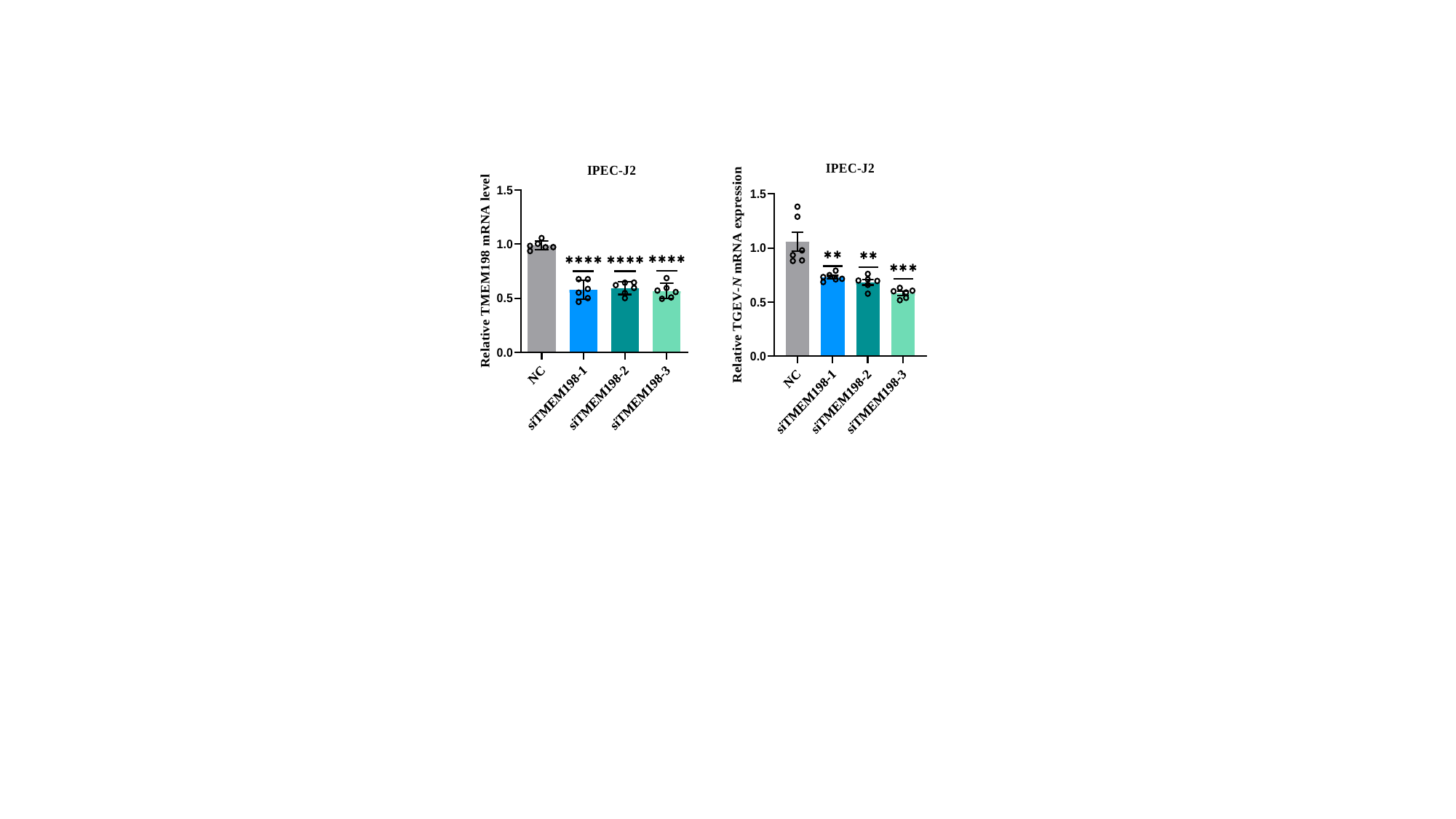

Supplement: S1 Fig — Left panel, siRNA-mediated knockdown of TMEM198 in IPEC-J2 cells and confirmed by RT-qPCR. Right panel, IPEC-J2 cells were then infected with TGEV and viral replication was analyzed by viral mRNA levels determined by qRT-PCR. (PPTX) [file ppat.1013211.s001.pptx]

## Slide 1
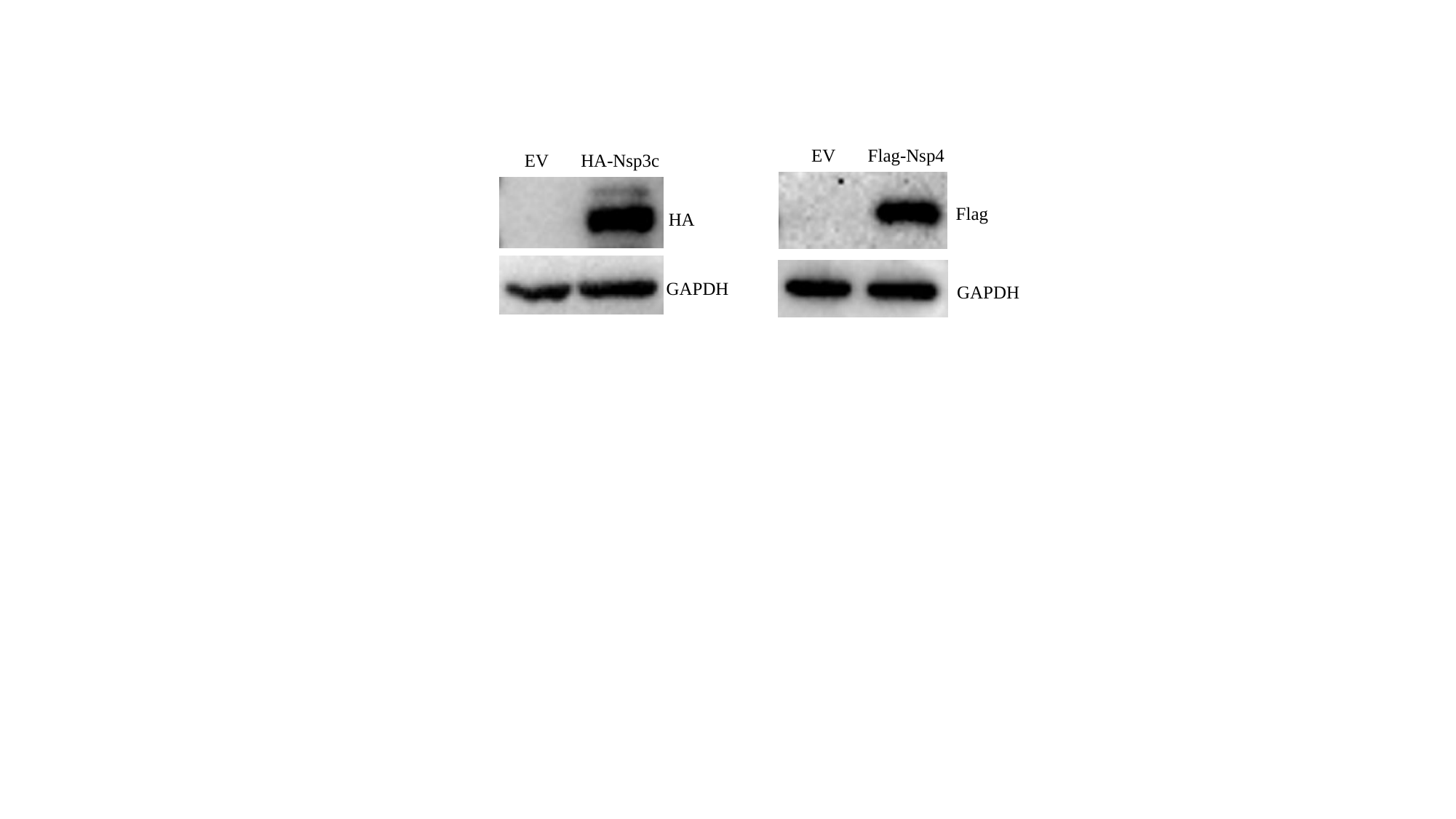

EV
Flag-Nsp4
EV
HA-Nsp3c
HA
GAPDH
Flag
GAPDH

Supplement: S2 Fig — (PPTX) [file ppat.1013211.s002.pptx]

## Slide 1
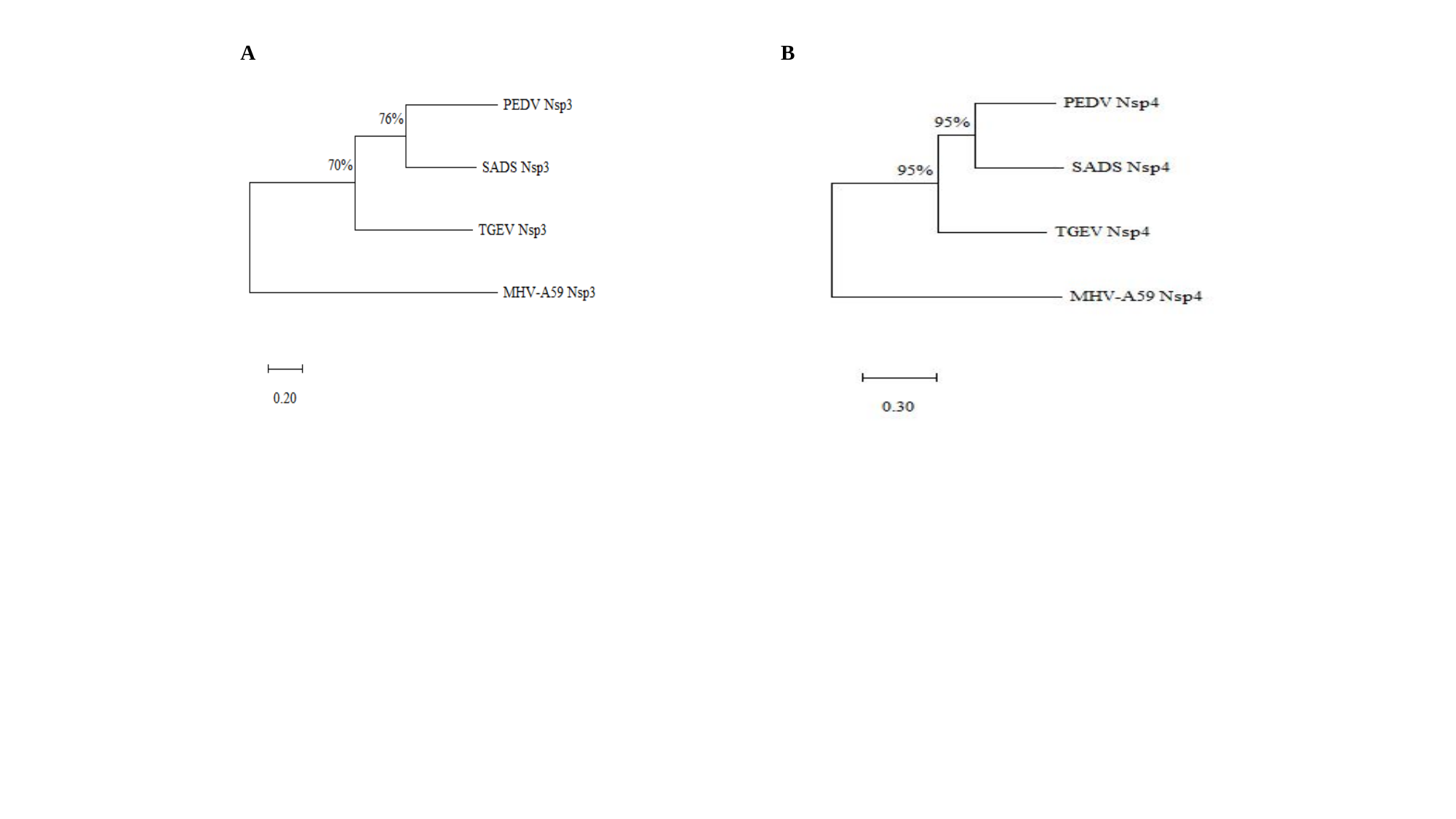

A
B

Supplement: S3 Fig — (A) Phylogenetic relationships between nsp3 of different coronavirus. (B) Phylogenetic relationships between nsp4 of different coronavirus. These phylogenetic trees were constructed using the maximum likelihood method in Mega11 software. (PPTX) [file ppat.1013211.s003.pptx]
